# Efficacy of Royal Guard, a new alpha-cypermethrin and pyriproxyfen treated mosquito net, against pyrethroid-resistant malaria vectors

**DOI:** 10.1038/s41598-020-69109-5

**Published:** 2020-07-22

**Authors:** Corine Ngufor, Abel Agbevo, Josias Fagbohoun, Augustin Fongnikin, Mark Rowland

**Affiliations:** 1 London School of Hygiene and Tropical Medicine (LSHTM), London, UK; 2 Centre de Recherches Entomologiques de Cotonou (CREC), Cotonou, Benin; 3 Pan African Malaria Vector Research Consortium (PAMVERC), Cotonou, Benin

**Keywords:** Malaria, Entomology

## Abstract

Royal Guard is a new insecticide-treated bed-net incorporated with a mixture of alpha-cypermethrin and pyriproxyfen (an insect growth regulator). We assessed its efficacy and wash-resistance in laboratory and experimental hut studies following WHO guidelines. Mosquitoes that survived exposure to the net were kept in separate oviposition chambers and observed for the reproductive effects of pyriproxyfen. In laboratory assays, Royal Guard induced > 80% mortality and > 90% blood-feeding inhibition of *An. gambiae* sl mosquitoes before and after 20 standardised washes and sterilised blood-fed mosquitoes which remained alive after exposure to the net. In an experimental hut trial against wild free-flying pyrethroid-resistant *An. gambiae* sl in Cové Benin, Royal Guard through the pyrethroid component induced comparable levels of mortality and blood-feeding inhibition to a standard pyrethroid-only treated net before and after 20 washes and sterilised large proportions of surviving blood-fed female mosquitoes through the pyriproxyfen component; Royal Guard induced 83% reduction in oviposition and 95% reduction in offspring before washing and 25% reduction in oviposition and 50% reduction in offspring after 20 washes. Royal Guard has the potential to improve malaria vector control and provide better community protection against clinical malaria in pyrethroid-resistant areas compared to standard pyrethroid-only LLINs.

## Background

Vector control is a major pillar in the control and prevention of malaria. It relies primarily on two core interventions; long-lasting insecticidal nets (LLINs) and indoor residual spraying (IRS)^1^. LLINs are particularly easier to deliver and highly cost-effective, providing both community protection^2,3^ and personal protection to the user. They are the most widely used public health intervention for malaria prevention and control and have thus contributed significantly to the remarkable reductions in malaria burden observed in endemic countries in the last two decades^4^. The World Health Organisation (WHO) recommends universal and continuous coverage with LLINs for all people at risk^1,5,6^.

Until very recently, bed-nets treated only with pyrethroids were the only class of LLINs covered by WHO policy recommendation. However, due to widespread and increasing intensity of pyrethroid resistance in malaria vectors^7^, the efficacy of pyrethroid-only LLINs is threatened hence new classes of LLINs which contain non-pyrethroid chemical compounds to which local vectors are susceptible are urgently needed^7^. With concerted efforts from chemical industries, academia, and product development partnerships, some new classes of LLINs are being developed for malaria vector control. Nets treated with a combination of pyrethroids and the synergist PBO (pyrethroid-PBO nets) have recently received conditional endorsement from WHO based on evidence from a cluster randomised trial in northern Tanzania demonstrating additional public health value of one type of pyrethroid-PBO net (Olyset Plus) compared to a pyrethroid-only LLIN product in an area with a moderate intensity of pyrethroid resistance partly conferred by monooxygenase-based resistance mechanism^8,9^. Pyrethroid-PBO LLINs are expected in theory to have an increased killing effect on malaria mosquitoes that express mixed-function oxidase based pyrethroid resistance mechanisms that are inhibited by the PBO in the net. However, they are unlikely to be effective everywhere as some vector populations have developed complex and multiple resistance mechanisms that may not be affected by the synergistic action of PBO^8,10^. In addition, due to wide variations in the design of pyrethroid-PBO nets associated with the technical complexities of applying PBO on bed-nets and ensuring its bioavailability and retention, it is unclear how most of the products in this LLIN class will perform under large scale field use. In any case, effective vector control and management of pyrethroid resistance in malaria vectors cannot rely on pyrethroid-PBO nets alone; other innovative and effective classes of LLINs treated with non-pyrethroids urgently need to be developed to increase the toolbox available to vector control programmes.

One novel class of insecticide-treated nets under consideration are nets treated with a combination of a pyrethroid and an insect growth regulator such as pyriproxyfen (pyrethroid-PPF nets). Pyriproxyfen is an insect juvenile hormone analogue which interferes with the reproduction and development of several insects, inhibiting embryogenesis and metamorphosis^11^. Pyriproxyfen has also been shown to inhibit oogenesis and sterilise adult female mosquitoes^12,13^. Adult mosquitoes exposed to pyriproxyfen, are therefore unable to contribute to the next generation of vectors leading to reductions in vector populations. Previous experimental hut studies investigating the efficacy of a pyrethroid-PPF net treated with a mixture of permethrin and pyriproxyfen (Olyset Duo) against wild pyrethroid-resistant malaria vectors in Benin and Cote D’Ivoire demonstrated the capacity of such nets to provide personal protection through the excito-repellent property of the pyrethroid while sterilising female mosquitoes which survive exposure to the net owing to pyrethroid resistance^14,15^. In a more recent community randomised controlled trial conducted in Burkina Faso, Olyset Duo provided improved protection against clinical malaria compared to pyrethroid-only LLINs in an area of intense transmission of *Plasmodium falciparum* malaria and high pyrethroid-resistant malaria vectors^16^.

Royal Guard is a new pyrethroid-PPF net developed by Disease Control Technologies, USA. It is treated with a mixture of alpha-cypermethrin and pyriproxyfen, both incorporated into the monofilament yarn during the extrusion process. In this study, we performed laboratory and experimental hut studies at the CREC/LSHTM GLP-compliant Facility in Benin to assess the efficacy and wash-resistance of Royal Guard against pyrethroid-resistant malaria vectors in Southern Benin as part of a submission dossier for WHO pre-qualification. The studies were conducted following guidelines established under the former WHO pesticide evaluation scheme (WHOPES)^17^. Royal Guard was compared to a standard PQ-listed pyrethroid-only LLIN and a pyriproxyfen-only net in both laboratory and experimental hut studies.

## Materials and methods

Royal Guard is a 120 dernier net made from a proprietary blend of high-density polyethylene (HDPE) and linear low-density polyethylene (LLDPE) treated with a mixture of alpha-cypermethrin and pyriproxyfen, both incorporated into the monofilament yarn during the extrusion process. Both insecticides are applied at the same concentration of 5.5 g/kg (225 ± 56.5 mg/m^2^).

### Study design and outcome measures

To be considered a long-lasting insecticidal net, Royal Guard would be expected to retain its biological efficacy over 20 standardised washes in both laboratory and experimental hut studies^17^. The wash-resistance of the net was thus assessed under laboratory conditions in WHO cone bioassays and tunnel tests using laboratory-maintained strains of *An. gambiae* sensu lato and in experimental hut trials against wild free-flying pyrethroid-resistant *An. gambiae* sensu lato in Cové, southern Benin. Efficacy was measured in terms of the capacity of Royal Guard to induce mortality, prevent blood-feeding and sterilise mosquitoes before washing and after several washes. After exposure at each level of experimentation, mosquitoes were initially observed for blood-feeding inhibition, excito-repellency and mortality (acute insecticidal effects) after which the survivors from each treatment were held in individual oviposition chambers and monitored for reproductive effects. The oviposition chambers which constituted netted plastic cups containing about 50 ml of freshwater were monitored daily for evidence of egg-laying, and the number of eggs laid by each female mosquito was recorded for up to 9 days. A pinch of larval food was added to any chamber that contained eggs, and the numbers of larvae that hatched were recorded after another 4 to 6 days.

The following outcome measures were used to assess the reproductive effects of the different nets on surviving female mosquitoes:

1. *% reduction in oviposition rate* The reduction in the proportion of females ovipositing for a given treatment compared to the control. This was calculated as follows:
  $$\frac{100(Oc - Ot)}{{Oc}}$$
  where *Oc* is the proportion of surviving blood-fed females from the control which laid eggs while *Ot* is the proportion of surviving blood-fed females from a given treatment which laid eggs.
2. *% reduction in fecundity* The reduction in the number of eggs per surviving blood-fed female for a given treatment relative to the control. This was calculated as follows:
  $$\frac{100(Ec - Et)}{{Ec}}$$
  where *Ec* is the mean number of eggs per surviving blood-fed female observed in the control while *Et* is the mean number of eggs per surviving blood-fed female observed in a given treatment.
3. *% reduction in offspring* the percentage reduction in the number of larvae per surviving blood-fed female observed for a given treatment relative to the control. This was calculated as follows:
  $$\frac{100(Lc - Lt)}{{Lc}}$$
  where *Lc* is the mean number of larvae per surviving blood-fed female observed in the control while *Lt* is the mean number of larvae per surviving blood-fed female observed in a given treatment.

### Laboratory bioassays

WHO cone bioassays and tunnel tests were conducted on net pieces of Royal Guard to assess its regeneration time, wash-resistance and efficacy against susceptible and resistant strains of *An. gambiae* sensu lato under controlled laboratory conditions. Royal Guard was compared to Royal Sentry (Disease Control Technologies, USA), a PQ-listed pyrethroid-only net incorporated only with alpha-cypermethrin at 5.8 g/Kg (191 mg/m^2^), and a pyriproxyfen-only incorporated net developed by DCT (Disease Control Technologies, LLC, USA) to the same technical specifications as Royal Guard. Net pieces measuring 25 × 25 cm were used for laboratory bioassays and these were obtained from four random whole nets from 2 production batches of each net-type^17^. An untreated control net was also tested as a negative control.

#### Regeneration time studies and net washing

Regeneration time is the time required for the insecticide efficacy of an LLIN to reach a plateau after washing and it is the minimum time that must be applied between successive washes in LLIN wash-resistance studies^17^. To assess regeneration time of Royal Guard, netting pieces (measuring 25 × 25 cm) were tested in WHO cone bioassays before washing and then washed 3 successive times and tested at daily intervals using the susceptible *An. gambiae* Kisumu strain. A total of 80 blood-fed 2-5 days old mosquitoes were exposed for 3 min to net pieces of each net type on each day of testing in replicates of 5 mosquitoes per cone. Regeneration of alpha-cypermethrin in Royal Guard was assessed by measuring knockdown of mosquitoes 1 h after exposure and mortality 24 h after each day of testing while the regeneration of pyriproxyfen was assessed by holding mosquitoes which survived exposure to PPF Net in individual oviposition chambers and observing for reproductive effects. To eliminate the confounding effects of the pyrethroid, the regeneration of pyriproxyfen was assessed on PPF Net as a proxy for Royal Guard. Twenty-eight net pieces of each net type measuring 25 × 25 cm were washed 1, 3, 5, 10, 15, 20 and 25 times in batches of 4 pieces per wash point at time intervals determined from the regeneration time studies. The washing process followed WHOPES guidelines^17^. Net samples were washed for 10 min using standardized soap solution (Savon de Marseille at 2 g per litre of deionized water) in shaker water baths set at 155 movements per minute and 30 °C. The samples were rinsed twice for 10 min in clean water under the same conditions as above and stored in an incubator at 30 °C and 75–85% relative humidity between washes and between tests.

#### WHO wash-resistance cone bioassays

WHO cone bioassays were performed with blood-fed 2–5 days old mosquitoes of the susceptible *An. gambiae* Kisumu and pyrethroid-resistant *An. gambiae* sl Cové. Mosquitoes of each strain were exposed for 3 min to net pieces of each net-type washed 0, 1, 3, 5, 10, 15, 20 and 25 times in replicates of 5 mosquitoes per cone. Four replicate netting pieces per wash point of each net type were tested while only 2 were tested for the control. All cone bioassays were carried out at 27 ± 2 °C and 75 ± 10% RH.

A total of approximately 160 susceptible *An. gambiae* Kisumu and 60 pyrethroid-resistant *An. gambiae* sl Cové blood-fed mosquitoes were tested per wash point of each net-type. Knockdown was recorded 1 h after exposure and mortality 24 h later. Only surviving blood-fed mosquitoes were held in individual oviposition chambers and observed for reproductive effects as described earlier. Unfed mosquitoes of both strains were also tested in the wash-resistance cone bioassays but these were not assessed for reproductive effects because they failed to take a blood meal after exposure and many were killed during or immediately after the feeding process leaving too few blood-fed survivors (2–4 mosquitoes) to assess for reproductive effects.

#### WHO wash-resistance tunnel tests

The tunnel test is an experimental chamber with an animal bait that allows expression of the behavioural interactions that occur between free-flying mosquitoes and bed nets during host-seeking. Tunnel tests were performed on net pieces of each net type washed 0 and 20 times using susceptible *An. gambiae* Kisumu and pyrethroid-resistant *An. gambiae* sl Cové mosquitoes. For each strain, each net type and each wash point, 3 net pieces were tested in 3 separate tunnels with ~ 80 mosquitoes per tunnel. The tunnel test consists of a square glass cylinder (25 cm high, 25 cm wide, 60 cm in length) divided into two sections by a netting frame fitted into a slot across the tunnel. In one of the sections, a guinea pig was kept unconstrained in a small cage, and in the other section, ~ 80 unfed female mosquitoes aged 5–8 days were released at dusk and left overnight. The net pieces were deliberately holed with nine 1-cm holes to give an opportunity for mosquitoes to penetrate the animal baited chamber for a blood meal; an untreated net sample served as the control. The tunnels were kept overnight in a dark room at 25–29 °C and 75–85% RH. The next morning, the numbers of mosquitoes found alive or dead, fed or unfed, in each section were scored. Live mosquitoes were provided with 10% glucose solution and delayed mortality recorded after 24 h. Blood-fed mosquitoes which remained alive after 24 h were assessed for sterilizing effects of pyriproxyfen as described earlier. The guinea pigs used in this study were kept following institutional guidelines for animal care.

### Experimental hut trial

#### Study site and vector profile

The experimental hut study was performed at the CREC/LSHTM experimental hut station in a rice field in Cové (7.21′ N 2.34′ E), Southern Benin. The vector species consists of a mixture of *An. coluzzii* and *An. gambiae ss* with the latter occurring at lower proportions (~ 23%) mostly in the dry season. The vector population is resistant to pyrethroids used on insecticide-treated nets; > 90% survival was observed with all pyrethroids in WHO susceptibility cylinder bioassays performed during the trial. Resistance is mediated by high frequencies of the L1014 *kdr* frequency (> 90%) and over-expression of metabolic enzymes^18^. Seven experimental huts of the West African design were used for the study.

#### Experimental hut treatments

Royal Guard was compared to Duranet (Shobikaa Impex Ltd), a standard PQ-listed pyrethroid-only polyethylene net incorporated with alpha-cypermethrin at 5.8 g/kg and the pyriproxyfen-only net (PPF Net). Each net-type was tested unwashed and after 20 standardised washes. The following 7 treatments were thus assessed in the experimental hut trial:

1. Untreated control polyethylene net.
2. PPF Net (Pyriproxyfen-only) unwashed.
3. PPF Net (Pyriproxyfen-only) washed 20 times.
4. Duranet (alpha-cypermethrin-only) unwashed.
5. Duranet (alpha-cypermethrin-only) washed 20 times.
6. Royal Guard (alpha-cypermethrin + pyriproxyfen) unwashed.
7. Royal Guard (alpha-cypermethrin + pyriproxyfen) washed 20 times.

To simulate the conditions of a torn net, each net (including the control) was holed with 6 deliberate 4 cm × 4 cm holes (two holes on each large side and one on each small side) following WHOPES guidelines^17^. Net washing for the hut trial also followed WHOPES guidelines. The nets were placed in an aluminium bowl containing 10 l of water and 2 g/l of Savon de Marseille and washed for a total of 10 min. Washed nets were rinsed with clean water following the same procedure and dried horizontally in the shade and stored at ambient temperature between washes. The time interval applied between washes (regeneration time) was 1 day for Duranet and 3 days for PPF Net and Royal Guard as determined in the regeneration time bioassays.

#### Hut trial design

To avoid any potential bias due to hut position, the treatments were rotated through the 7 experimental huts every week of the trial using a randomised Latin Square Design which helped minimise carry-over effects between treatments. The hut trial lasted for 52 nights between November 2017 and February 2018. Data collection was done for 6 days within each week and on the 7th day, huts were thoroughly cleaned and aired in preparation for the next rotation cycle. Three replicate nets were tested per hut treatment and these were swapped every 2 days within each week of the trial. Seven (7) consenting human volunteer sleepers slept in the huts from dusk to dawn daily throughout the trial and to account for individual attractiveness to mosquitoes, they were rotated daily between the huts using a simple Latin Square Design. Mosquitoes were collected daily from the room, veranda trap and inside the net for each experimental hut and brought to the laboratory for identification and scoring. After immediate observations for blood-feeding, exophily and mortality (up to 24 h), live blood-fed female mosquitoes from each treatment were chambered individually and provided the opportunity to oviposit to assess reproductive effects as earlier described.

The following outcome measures were used to assess the efficacy of the treatments in the experimental huts.

For acute insecticidal effects:

- Hut deterrence estimated as the percentage reduction in numbers collected in a given treatment compared to the control.
- Exophily rates estimated from the proportions of mosquitoes collected from the verandas of all mosquitoes collected.
- Mortality estimated as the proportion of dead mosquitoes after 24 h of all mosquitoes collected.
- Blood-feeding inhibition estimated as the proportional reduction in blood-feeding in huts with insecticide-treated nets relative to controls with untreated nets.

For reproductive effects of the different nets on surviving blood-fed female mosquitoes from each experimental hut, the outcome measures were % reduction in oviposition rate, % reduction in fecundity and % reduction in offspring in a given treatment relative to the control as earlier described.

### Chemical analysis of insecticide content

To investigate within- and between-net variation of the active ingredients in Royal Guard (pyriproxyfen and alpha-cypermethrin) and their respective wash-resistance indexes, chemical analysis were performed at reference laboratories (CRA-W of Gembloux, Belgium and IIBAT India) with net pieces (25 × 25 cm) from the laboratory bioassays and experimental hut trials. At CRA-W, the chemical content of alpha-cypermethrin and pyriproxyfen in net pieces of Royal Guard from the laboratory bioassays were assessed using the CIPAC method 5107/R (extension of CIPAC 454/LN/M/3.2). This method involves the extraction of alpha-cypermethrin and pyriproxyfen in a water bath at 85–90 °C for 45 min with heptane in presence of dicyclohexyl phthalate as internal standard and determination by gas chromatography with flame ionisation detection (GC-FID). At IIBAT, alpha-cypermethrin content in net pieces (25 cm × 25 cm) obtained from the nets used in the experimental hut trial was determined by GC-FID (CIPAC 454/LN/M/3.2) while pyriproxyfen content was determined by reverse-phase high-performance liquid chromatography (HPLC) using UV detector, at a detection wavelength of 254 nm with dicyclohexyl phthalate as internal standard (CIPAC 715/TC/M/3).

The identity of the active ingredients was established by comparison with the equivalent authentic standard. At each laboratory, each sample was analysed separately and the average content per treatment was obtained. The wash-resistance index was calculated according to WHO guidelines^17^ as indicated below:

$${\text{Wash resistance index }} = { 1}00 \times {\text{n}}\surd ({\text{t}}{\mathbf{n}}/{\text{t}}{\mathbf{0}}) \, \left( {\text{free migration stage behaviour}} \right)$$

where t**n** = total active ingredient content after n washing cycles t**0** = total active ingredient content before washing n = number of washes.

### Statistical analysis

Generalised linear models (GLM) were used to analyse proportional data (knockdown, mortality, blood-feeding, exiting, oviposition rate of surviving blood-fed females) from the cone, tunnel and experimental hut data, comparing different net types and the number of washes. A separate model was fitted for each outcome. Each model included random effects to account for sources of variations related to different replicates and different days of testing in the laboratory bioassays and between the seven huts, between the seven sleepers and between the weeks of the experimental hut trial. Differences in numbers of eggs laid per surviving female in each treatment were compared using independent t-tests. All analyses were performed using STATA version 11.1 Texas USA.

### Ethical approval

Ethical approval for the study was obtained from the national ethics review board of the Ministry of Health in Benin (CNERS No. 39) and from the institutional ethics review board of the Centre de Recherche Entomologique de Cotonou (CREC), Benin. Written informed consent was obtained from each volunteer sleeper before they participated in the study. A stand-by nurse was available to the sleepers throughout the study to assess any cases of fever. Any sleepers testing positive for malaria were withdrawn from the study and fully treated. Sleepers were also offered chemoprophylaxis before they participated in the trial. Animals (guinea pigs) used in tunnel tests and mosquito feeding were kept in line with institutional guidelines and standard operating procedures for animal care. All methods were carried out following relevant guidelines and regulations.

## Results

### Laboratory studies

#### Regeneration time of Royal Guard

**Figure 1:**
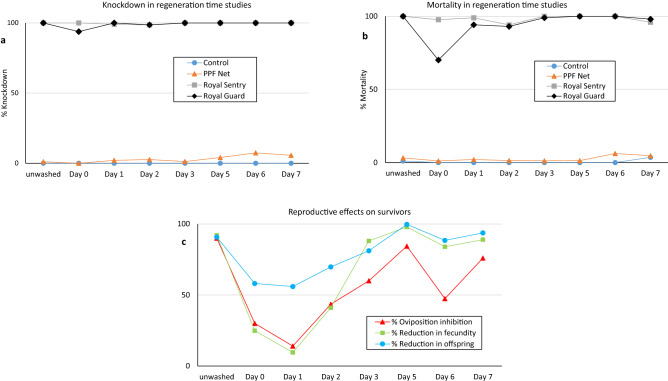
Knockdown (**a**), mortality (**b**) and reproductive effects (**c**) on susceptible *An. gambiae* Kisumu in regeneration time cone bioassay studies.

The results from the regeneration time bioassays with pyrethroid susceptible *An. gambiae* Kisumu is summarised in Fig. 1. The knockdown and mortality effect of alpha-cypermethrin in Royal Guard were 100% before washing, reduced slightly immediately after washing (95% knockdown and 70% mortality on Day 0) but returned to the pre-wash level within 24 h post washing (Day 1), showing that the biological efficacy of the insecticide regenerated within 1 day (Fig. 1a,b). Because mortality rates were mostly 100% after exposure to Royal Guard, the results on reproductive effects obtained with PPF Net were used as a proxy for the regeneration of pyriproxyfen in Royal Guard since both nets were developed with the same technical specifications. All three reproductive suppression parameters (reduction in oviposition, fecundity and offspring) were very high before washing (> 90%), reduced significantly up to 24 h after washing but returned to a somewhat steady level from Day 3 and beyond (Fig. 1c). The regeneration time of pyriproxyfen in Royal Guard was thus 3 days. According to WHOPES guidelines, the regeneration time for mixture LLINs should be based on the longer regeneration between the active ingredients involved. The Royal Guard nets were washed using a 3 days regeneration time interval for subsequent wash-resistance studies. The data also confirmed the regeneration time of Royal Sentry to be 1 day.

#### Wash resistance cone bioassay results

**Figure 2:**
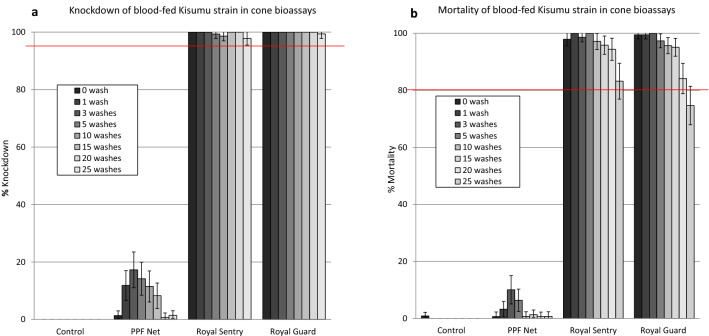
Knockdown (**a**) and mortality (**b**) rates of blood-fed susceptible *An. gambiae* Kisumu in wash resistance cone bioassays. Mosquitoes were blood-fed before exposure to the nets. Error bars represent 95% confidence intervals. The red line indicates WHO cut-off criteria for efficacy in cone bioassays.

**Figure 3:**
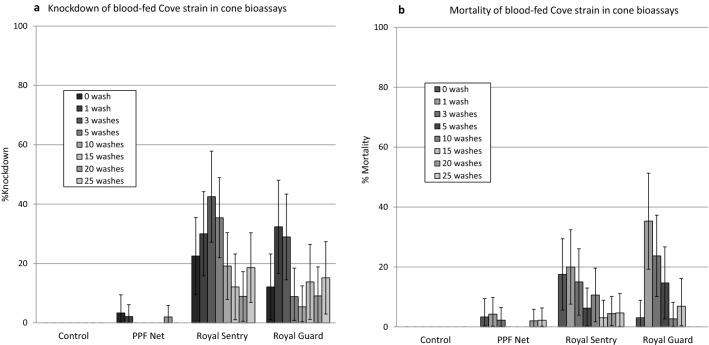
Knockdown (**a**) and mortality (**b**) rates of blood-fed pyrethroid-resistant *An. gambiae* sl Cové in wash resistance cone bioassays. Mosquitoes were blood-fed before exposure to the nets. Error bars represent 95% confidence intervals.

Knockdown and mortality rates of blood-fed susceptible *An. gambiae* Kisumu in wash resistance cone bioassays were 0% with the control and < 20% with PPF Net at all wash points (Fig. 2). With Royal Sentry and Royal Guard, knockdown rates were > 95% at all wash points tested (Fig. 2a). Mortality of susceptible *An. gambiae* Kisumu with both net types were > 80% for up to 25 washes with Royal Sentry and up to 20 washes with Royal Guard (Fig. 2b). These results with the susceptible Kisumu strain showed that both nets meet WHO criteria for efficacy in wash-resistance cone bioassays with regards to the pyrethroid component. With pyrethroid-resistant *An. gambiae* sl Cové (blood-fed before exposure), knockdown and mortality rates were  < 40% across all wash points tested (Fig. 3) owing to high levels of pyrethroid resistance in the strain^18^.

The wash-resistance cone bioassay results achieved with unfed mosquitoes of both strains were very similar to what was achieved with blood-fed mosquitoes (Supplementary Fig. S1). Royal Sentry and Royal Guard again passed WHO criteria for both knockdown (> 95% for up to 20 washes) and mortality (> 80% for up to 20 washes) with the unfed susceptible Kisumu strain (Supplementary Fig. S1a) while very low knockdown and mortality (< 20%) was observed with unfed pyrethroid-resistant Cové strain (Supplementary Fig. S1b).

Due to high mortality rates achieved with the susceptible *An. gambiae* Kisumu strain (85–100%) in the wash-resistance cone bioassays, the impact of washing on the sterilising effects of pyriproxyfen in Royal Guard could only be assessed on the PPF Net as a proxy for Royal Guard. All three parameters for suppression of reproduction (reduction in oviposition, fecundity and offspring) were 100% when the net was unwashed (Table 1). Reduction in oviposition declined to 39% at 5 washes and remained at around this level for up to 25 washes while the reduction in fecundity and offspring remained high and showed very minimal variation as the number of washes increased. Hence the sterilising effects of pyriproxyfen in PPF Net and thus in Royal Guard was evident even after 25 washes. While the initial plan was to blood-feed surviving unfed pyrethroid-resistant mosquitoes after exposure in cone bioassays and observe them for reproductive effects, this was not done because the unfed survivors failed to take a blood meal post-exposure to the treated net samples or were killed during or immediately after feeding.

**Table 1:** Reproductive effects on surviving blood-fed susceptible *An. gambiae* Kisumu from wash-resistance cone bioassays.

	Control	PPF Net							
Wash point	Unwashed	Unwashed	1 wash	3 washes	5 washes	10 washes	15 washes	20 washes	25 washes
N observed	80	90	90	90	90	90	90	85	88
N females laying	58	0	0	8	40	27	41	44	44
N eggs	4,905	0	0	680	3,478	2,202	4,370	4,293	4,380
N larvae	2010	0	0	172	941	443	1,016	1,304	1589
% Ovipositing	73^a^	0^b^	0^b^	9^c^	44^de^	30^e^	46^d^	52^d^	50^d^
95% conf intervals	63–83	0–5	0–5	3–15	34–54	21–39	36–56	42–62	40–60
Hatch rate	41	–	–	25	27	20	23	30	36
Eggs/female laying	85	–	–	85	87	82	107	98	100
Egg/female observed	61	0	0	8	39	24	49	51	50
Larvae/female observed	25	0	0	2	10	5	11	15	18
% Oviposition inhibition	–	100	100	88	39	59	37	30	31
% Reduction in fecundity	–	100	100	88	37	60	21	18	19
% Reduction in offspring	–	100	100	92	58	80	55	39	28

Values along a row bearing the same letter label are not significantly different (GLM, P > 0.05).

The results on the reproductive effects on surviving blood-fed pyrethroid-resistant *An. gambiae* sl Cové from the cone bioassays is presented in Table 2. Oviposition rate in the control was low (8%). Nevertheless, the data showed that the reduction in oviposition, fecundity and offspring of mosquitoes which survived exposure to Royal Guard in the cone bioassays was 100% before washing and remained so for up to 20 washes. The PPF Net also induced significant reductions in oviposition, fecundity and offspring but this was more variable over the different wash points compared to Royal Guard. The oviposition inhibition achieved with Royal Sentry was 0% for most wash points tested and though some reduction in fecundity and offspring was achieved with Royal Sentry, this was generally lower than what was observed with the pyriproxyfen treated nets.

**Table 2:** Reproductive effects on surviving blood-fed pyrethroid-resistant *An. gambiae* sl Cové from wash-resistance cone bioassays treatment.

Treatment	Wash point	N observed	N Female laying	N eggs	N larvae	% Oviposition	Hatch rate	Eggs/female laying	Egg/females observed	Larvae/female observed	% Oviposition inhibition	% Reduction in fecundity	% Reduction in offspring
Control	0 wash	24	4	635	433	8	68	156	26	18			
PPF Net	0 wash	9	0	0	0	0	–	–	0	0	100	100	100
	1 wash	20	1	121	0	5	0	121	6	0	38	77	100
	3 washes	20	1	110	2	5	2	110	6	0	38	79	99
	5 washes	23	3	284	53	13	19	95	12	2	0	53	87
	10 washes	20	0	0	0	0	–	–	0	0	100	100	100
	15 washes	20	1	120	10	5	8	120	6	1	38	77	97
	20 washes	20	1	88	0	5	0	88	4	0	38	83	100
	25 washes	20	1	111	0	5	0	111	6	0	38	79	100
Royal Sentry	0 wash	19	2	232	199	11	11	116	12	10	0	53	42
	1 wash	12	2	377	189	17	50	189	31	16	0	0	13
	3 washes	14	3	307	116	21	38	102	22	8	0	16	54
	5 washes	18	1	154	112	6	73	154	9	6	31	67	65
	10 washes	20	2	160	66	10	41	80	8	3	0	69	82
	15 washes	12	1	100	38	8	38	100	8	3	0	68	82
	20 washes	17	1	120	100	6	14	120	5	6	26	82	67
	25 washes	20	2	192	177	10	15	96	10	9	0	63	51
Royal Guard	0 wash	20	0	0	0	0	–	–	0	0	100	100	100
	1 wash	22	0	0	0	0	–	–	0	0	100	100	100
	3 washes	26	0	0	0	0	–	–	0	0	100	100	100
	5 washes	16	0	0	0	0	–	–	0	0	100	100	100
	10 washes	20	0	0	0	0	–	–	0	0	100	100	100
	15 washes	16	0	0	0	0	–	–	0	0	100	100	100
	20 washes	20	0	0	0	0	–	–	0	0	100	100	100
	25 washes	22	2	138	66	9	48	69	6	3	0	76	83

#### Wash resistance tunnel test results

**Figure 4:**
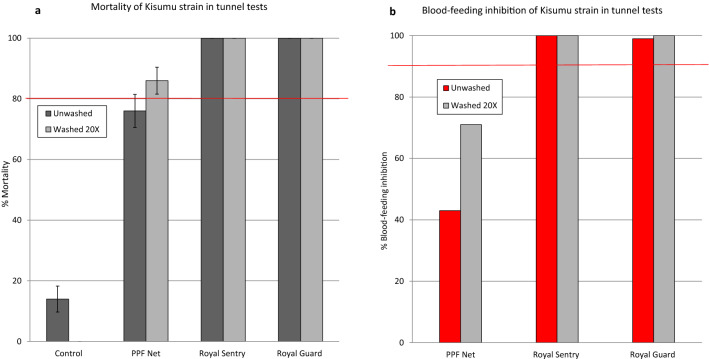
Mortality (**a**) and blood-feeding inhibition (**b**) of susceptible *An. gambiae* Kisumu in wash-resistance tunnel tests. Error bars represent 95% confidence intervals. The red line indicates WHO cut-off criteria for efficacy in tunnels.

Mortality with the susceptible Kisumu strain (Fig. 4) was 14% in the untreated control tunnels. Both Royal Sentry and Royal Guard killed all the *An. gambiae* Kisumu mosquitoes in the tunnels when unwashed and after 20 washes (100% mortality). Royal Guard, therefore, fulfilled WHO criteria for mortality in tunnel test (> 80%) after 20 washes. The PPF Net killed a high proportion of susceptible Kisumu in the tunnels when unwashed (76%) and after 20 washes (86%). Blood feeding inhibition of the susceptible Kisumu strain in tunnel tests was > 98% with Royal Sentry and Royal Guard when unwashed and after 20 washes (Fig. 4b). Royal Guard, therefore, showed no loss of blood-feeding inhibition after 20 washes and thus fulfilled WHO efficacy criteria for blood-feeding inhibition in tunnel tests (> 90% after 20 washes). The PPF Net provided some blood-feeding inhibition against the susceptible Kisumu strain (43% with unwashed and 71% after 20 washes) but this was lower than what was achieved with the pyrethroid treated nets (> 95%).

**Figure 5:**
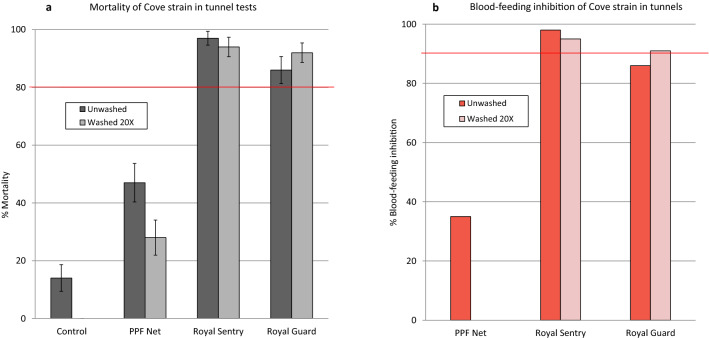
Mortality (**a**) and blood-feeding inhibition (**b**) of pyrethroid-resistant *An. gambiae* sl Cové in wash-resistance tunnel tests. Error bars represent 95% confidence intervals. The red line indicates WHO cut-off criteria for efficacy in tunnels.

Mortality rates of the pyrethroid-resistant *An. gambiae* sl Cové strain (Fig. 5a) were very high with Royal Sentry (97% with unwashed and 94% after 20 washes) and Royal Guard (86% with unwashed and 92% after 20 washes). This was unexpected considering the high levels of pyrethroid resistance in this strain. Perhaps alpha-cypermethrin in both nets was more toxic to the pyrethroid-resistant strain in the tunnel test owing to the longer exposure time in tunnels. Nevertheless, the mortality rates achieved were less than what was achieved with the susceptible Kisumu strain. The PPF Net also induced some mortality against the pyrethroid-resistant Cové strain in the tunnels (47% with unwashed and 28% after 20 washes) but this was also less than what was achieved against the susceptible Kisumu strain. With the pyrethroid-resistant Cové strain, Royal Sentry induced > 90% blood-feeding inhibition when unwashed and after 20 washes (Fig. 5b). With Royal Guard, blood-feeding inhibition was 86% when unwashed and 91% after 20 washes, hence the net also met WHO efficacy criteria after 20 washes (≥ 80% mortality and/or ≥ 90% blood-feeding inhibition) against the pyrethroid-resistant Cové strain.

The proportions of surviving blood-fed susceptible Kisumu mosquitoes from the control tunnel which laid eggs was 69% (24/35) producing an average of 62 eggs and 27 larvae per female observed (Table 3). None of the blood-fed susceptible Kisumu mosquitoes exposed to the unwashed PPF Net in tunnels laid eggs, hence PPF Net reduced their oviposition, fecundity and offspring by 100% compared to the control. This effect remained substantial with PPF Net washed 20 times; all reproductive parameters compared to the control reduced by 79–89%. No live susceptible *An. gambiae* Kisumu mosquitoes were collected in tunnel tests with Royal Guard and Royal Sentry (mortality was 100%) hence it was not possible to assess the wash-resistance of pyriproxyfen in Royal Guard using this strain. However, because PPF Net has the same specification as Royal Guard (but lacking alpha-cypermethrin), the wash-resistance tunnel tests results of pyriproxyfen in PPF Net applies to Royal Guard.

**Table 3:** Reproductive effects on surviving blood-fed susceptible *An. gambiae* Kisumu from tunnel tests.

	Control	PPF Net	
		Unwashed	Washed 20 ×
N alive blood-fed observed	35	31	19
N laying	24	0	5
%Laying	69	0	26
N eggs	2,176	0	204
N larvae	948	0	54
Hatch rate (%)	44	–	26
Eggs/female laying	91	–	41
Egg/females observed	62	0	11
Larvae/female observed	27	0	3
%Oviposition inhibition	–	100	79
%Reduction in fecundity	–	100	82
%Reduction in offspring	–	100	89

The proportions of pyrethroid-resistant *An. gambiae* sl Cové from the control tunnel which laid eggs was very low (3%, n = 132) and this could be attributed to the fact that the strain was recently colonised and was thus less adapted to laboratory rearing. The results on the reproductive effects on the resistant strain were therefore difficult to interpret. Besides, due to the unusually high mortality rates (86–97%) and blood-feeding inhibition (86–98%) achieved with Royal Sentry and Royal Guard against the pyrethroid-resistant Cové strain in the tunnel tests, no surviving blood-fed females exposed to Royal Sentry and Royal Guard were found.

### Experimental hut trial results

#### Mosquito entry and exiting rates

A total of 8,123 wild free-flying female pyrethroid-resistant *An. gambiae* sl were collected in the experimental huts during the trial (Table 4). Mosquito deterrence was low with PPF Net (10.5% unwashed and 7% when washed) and Royal Guard (3.9% unwashed and 9.5% when washed) but higher with Duranet (47.6% unwashed and 38.7% when washed). This could perhaps be due to the slightly higher dose of alpha-cypermethrin in Duranet inducing higher levels of mosquito repellency from the hut. Exiting rates did not differ significantly between Royal Guard and Duranet when unwashed (58% with Royal Guard and 53% with Duranet; P = 0.2) and after 20 washes (47% with Royal Guard and 47% with Duranet, P = 0.81).

**Table 4:** Entry and exiting rates of wild free-flying pyrethroid-resistant *An. gambiae* sl in experimental huts in Cové, Benin.

	Control net	PPF Net		Duranet		Royal Guard	
No of washes	–	0	20	0	20	0	20
Total females caught	1,394	1,247	1,296	731	854	1,339	1,262
Average catch per night	26	23	24	14	16	25	23
% Deterrence	–	10.5	7	47.6	38.7	3.9	9.5
Total exiting	603	494	386	391	400	775	589
% Exiting	43^a^	40^a^	30^b^	53^d^	47^c^	58^d^	47^c^
95% conf interval	(41–46)	(37–42)	(27–32)	(50–57)	(43–50)	(55–61)	(44–49)

Values along a row bearing the same letter label are not significantly different at 5% level (GLM, P > 0.05).

#### Blood feeding inhibition

**Figure 6:**
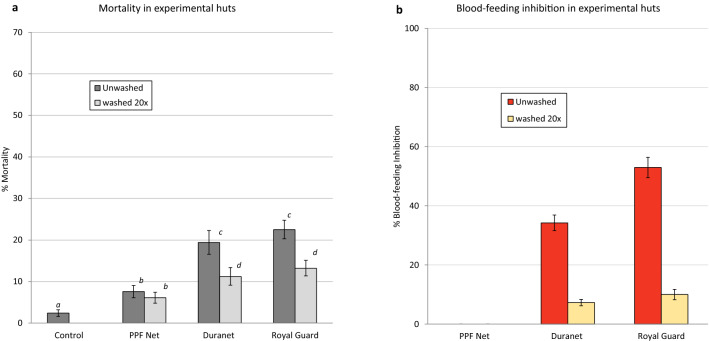
Mortality (**a**) and blood-feeding inhibition (**b**) of wild free-flying pyrethroid-resistant *An. gambiae* sl in experimental huts in Cové, Benin. Bars with the same letter label are not significantly different; GLM, P > 0.05. Error bars represent 95% CI.

Blood feeding rates with the control (untreated net) was 50% (Supplementary Table S1). PPF Net did not induce any blood-feeding inhibition both when unwashed and after 20 washes (Fig. 6b). Blood-feeding inhibition with unwashed Duranet was 34% but this declined significantly when the net was washed 20 times (7%, P < 0.001). Royal Guard induced significantly higher blood-feeding inhibition than Duranet when unwashed (53% with Royal Guard and 34% with Duranet, P < 0.05) and similar levels of blood-feeding inhibition when washed 20 times (10% with Royal Guard and 7% with Duranet, P > 0.05). Royal Guard therefore performed as well as Duranet in its capacity to inhibit mosquito feeding when washed and after 20 washes.

#### Mortality rates

Mortality with the control was 2.4% (Fig. 6a, Supplementary Table S1). Mortality with PPF Net was low; 7.6% when unwashed and 6.1% when washed. Royal Guard induced similar mortality rates to Duranet when unwashed (22.5% with Royal Guard vs. 19.4% with Duranet, P = 0.29) and after 20 washes (13.2% with Royal Guard and 11.2% with Duranet, P = 0.82).

#### Reproductive effects on surviving mosquitoes from experimental huts

A total of 2,938 blood-fed wild pyrethroid-resistant *An. gambiae* sl mosquitoes which remained alive from the experimental huts were held in individual oviposition chambers and observed for reproductive effects. Results are presented in Tables 5 and 6.

**Table 5:** Reduction in oviposition rate for surviving wild free-flying blood-fed pyrethroid-resistant *An. gambiae* sl mosquitoes from experimental huts in Cové, Benin.

Net type	No of washes	No. of females observed	No. of females laying eggs	% Blood-fed females laying eggs	95% CI	% Reduction in oviposition
Control	–	506	177	35^a^	(31–39)	–
PPF Net	0	546	26	5^b^	(3–7)	86
	20	719	174	24^c^	(21–27)	31
Duranet	0	157	55	35^a^	(28–43	0
	20	265	90	34^a^	(28–40)	3
Royal Guard	0	238	14	6^b^	(3–9)	83
	20	507	134	26^c^	(22–30)	25

Values in the same column bearing the same letter label are not significantly different at 5% level (GLM, P > 0.05).

**Table 6:** Reduction in fecundity and offspring of surviving wild free-flying blood-fed pyrethroid-resistant *An. gambiae* sl from experimental huts in Cové, Benin.

Net type	No of washes	Total eggs laid	Eggs per females observed	95% CI	% Reduction in fecundity	Hatch rate	Larvae per female observed	% Reduction in offspring
Control	–	20,634	41^a^	36–46	–	30	12	–
PPF Net	0	2,433	4^b^	2–6	89	16	1	94
	20	21,059	29^c^	25–34	28	21	6	50
Duranet	0	7,094	47^a^	36–57	− 11	28	13	− 6
	20	10,434	40^a^	34–46	3	34	13	− 12
Royal Guard	0	1,265	5^b^	2–8	87	10	0.5	95
	20	15,451	30^c^	25–36	25	20	6	50

Values in the same column bearing the same letter label are not significantly different at 5% level (GLM, P > 0.05).

Oviposition rates of surviving wild blood-fed pyrethroid-resistant *An. gambiae* sl from the experimental huts with Duranet were 35% when unwashed and 34% after 20 washes and this did not differ significantly from the control (35%, P > 0.05) (Table 5). Oviposition rates were significantly lower with PPF Net and Royal Guard especially when unwashed (4.8% and 6% respectively, P < 0.05). Oviposition inhibition was thus very high with PPF Net and Royal Guard when unwashed (86% and 83% respectively). Although this effect declined after 20 washes (31% with PPF Net and 25% with Royal Guard), it remained substantially higher than what was observed with Duranet (0% when unwashed and 3% after 20 washes).

The effects on the fecundity (eggs laid per female observed) and offspring (larvae per female observed) are presented in Table 6. An average of 41 eggs were produced per surviving female from the control and this did not differ significantly from Duranet both unwashed and after 20 washes (P > 0.05). A significantly lower number of eggs per female were observed with PPF Net and Royal Guard when unwashed (4 and 5 respectively) and after 20 washes (29 and 30 respectively) compared to the control and Duranet (P < 0.05). This resulted in high levels of reduction in fecundity compared to the control with the PPF treated nets when unwashed (89% with PPF Net and 87% with Royal Guard); this effect, however, declined substantially after 20 washes (28% with PPF Net and 25% with Royal Guard). The overall reduction in offspring with PPF Net and Royal Guard relative to the control showed a similar trend; 94% and 95% respectively when unwashed and both 50% after 20 washes. Duranet did not induce any reduction in fecundity or offspring compared to the control both when unwashed and after 20 washes. Royal Guard was therefore superior to Duranet in its capacity to sterilise and reduce the offspring of surviving wild pyrethroid-resistant blood-fed *An. gambiae* sl mosquitoes both before washing and after 20 washes in the experimental hut trial.

### Chemical analysis results of samples from laboratory and field studies

Analysis of 20 net pieces obtained from 4 different randomly selected unwashed whole Royal Guard nets (1 per side) revealed an average a.i. content of 5.1–5.6 g/kg for alpha-cypermethrin and 4.8–5.2 g/kg for pyriproxyfen demonstrating that the nets comply with the target dose of 5.5 g/kg ± 25% for both a.i.s. The within-net variation expressed as the relative standard deviation (RSD) of the a.i. content found on 5 net pieces obtained from the same net ranged from 5.1 to 12.4% for alpha-cypermethrin and 4.2% to 14% for pyriproxyfen showing an acceptable homogeneity of the distribution of both a.i.s over the net. The RSD of a.i. content on net pieces obtained from 4 different Royal Guard nets was 3.7% for alpha-cypermethrin and 4.3% for pyriproxyfen also showing acceptable between-net variation.

**Figure 7:**
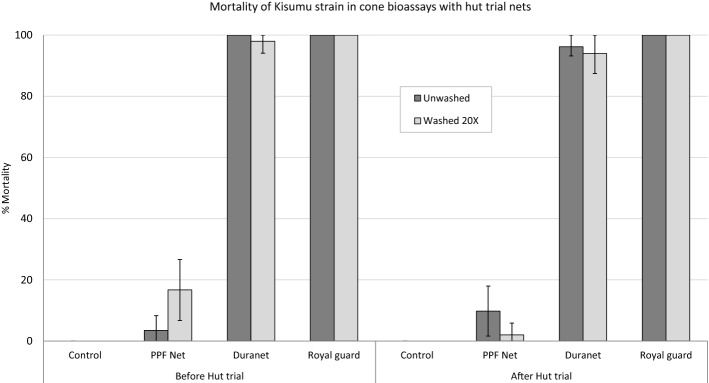
Mortality (%) of susceptible *An. gambiae* Kisumu in cone bioassays with hut nets before and after the experimental hut trial in Cové, Benin. Error bars represent 95% confidence intervals.

The chemical content of net pieces washed in the laboratory 1, 3, 5, 10, 15, 20 and 25 times showed that washing induced a faster decline in pyriproxyfen content (54.3% retention after 25 washes) compared alpha-cypermethrin (71.9% retention after 25 washes) (Table 7). Wash resistance index was thus higher with alpha-cypermethrin (98.7%) compared to pyriproxyfen (97.8%). By contrast, Royal Guard nets from the experimental huts showed a higher wash-resistance index (97–98%) with pyriproxyfen compared to alpha-cypermethrin (94–95%). The washing method and handling of nets for laboratory assays and experimental hut trials are different and may have affected the retention of both insecticides. However, cone bioassays performed with Royal Guard net pieces obtained from nets used in the hut trial showed no decline in mortality after 20 washes both before and after the hut trial (Fig. 7).

**Table 7:** Wash retention of alpha-cypermethrin and pyriproxyfen in Royal Guard.

Active ingredient (a.i.)	No. of washes	Actual a.i. content (g/kg)*	Relative standard deviation (RSD)	Actual a.i. retention (% of wash 0)
Alpha-cypermethrin	0	5.2	4.5	–
	1	5.0	5.3	96.3
	3	4.9	1.9	94.7
	5	4.7	2.8	89.9
	10	4.3	5.0	82.9
	15	4.1	2.1	78.6
	20	4.0	2.8	76.2
	25	3.7	4.2	71.9
Pyriproxyfen	0	4.3	5.1	–
	1	3.8	3.4	89.3
	3	3.5	2.1	80.3
	5	3.3	2.7	75.5
	10	3.0	3.6	69.7
	15	2.7	1.4	62.7
	20	2.5	7.1	57.5
	25	2.3	2.8	54.3

*a.i.* active ingredient.

*Each value is the mean a.i. content in 4 net pieces.

## Discussion

This study demonstrates in both laboratory and experimental hut trials, the superiority of Royal Guard over pyrethroid-only LLINs in terms of its ability to sterilise surviving pyrethroid-resistant mosquitoes through the pyriproxyfen component while also inducing mortality and preventing mosquito feeding through the effect of alpha-cypermethrin. This confirms previous findings with Olyset Duo, a permethrin-PPF net which was formerly evaluated in similar studies by this same facility^14,15^ and others^19,20^, demonstrating that the addition of an insect growth regulator to pyrethroids on bed-nets is an important innovation. Surviving pyrethroid-resistant mosquito vectors which are sterilised by pyriproxyfen in the net do not contribute to the next generation of vectors hence continuous community-wide use of pyrethroid-PPF nets should lead to significant reductions in vector densities over time thus reducing malaria transmission.

As a new candidate LLIN, Royal Guard is expected to retain its biological efficacy after 20 standardised washes in laboratory and experimental hut studies^17^ before community-scale application. Twenty washes are indicated by WHO as a proxy for its ability to withstand multiple washes under operational use over a 3-year life span. In this study, Royal Guard fulfilled all existing WHO criteria for efficacy at all levels of evaluation in terms of mosquito knockdown, mortality and blood-feeding inhibition thus demonstrating the wash-resistance of alpha-cypermethrin in the net. Royal Guard was granted interim WHO recommendation as a long-lasting pyrethroid net based on these findings. Unlike for pyrethroids, clear testing procedures and efficacy criteria for pyriproxyfen on bed-nets are yet to be specified in WHO guidelines since these guidelines were originally tailored towards the evaluation of pyrethroid-only LLINs. In laboratory bioassays, pyrethroid susceptible and resistant mosquitoes which survived exposure to Royal Guard and the pyriproxyfen-only net which was developed to the same technical specification as Royal Guard were sterilised significantly for up to 20 washes. Reproductive suppression of wild pyrethroid-resistant mosquito survivors from experimental huts with unwashed Royal Guard and PPF Net was initially very high and then declined by 50–70% after 20 washes. Royal Guard was therefore superior to the pyrethroid-only LLIN in both laboratory bioassays and experimental huts in terms of its additional effect on mosquito reproduction even after 20 washes.

For all new classes of insecticide treated nets, WHO requires evidence of improved epidemiological effect over standard pyrethroid-only LLIN^21^. A recent community randomised trial of Olyset Duo in a pyrethroid-resistant area in Burkina Faso demonstrated significant reductions in malaria vector densities and infectivity associated with community-wide use of the permethrin-PPF net compared to a pyrethroid-only net^16^. While the application dose of pyriproxyfen is lower in Royal Guard (5.8 g/kg) compared to Olyset Duo (10 g/kg), Royal Guard generally induced more substantial levels of reproductive suppression in laboratory bioassays and experimental hut studies compared to what we previously reported with Olyset Duo^14^. The sterilising effect of pyriproxyfen in Olyset Duo was completely lost in cone bioassays after only 5 washes contrary to Royal Guard which remained effective in suppressing mosquito reproduction for up to 20 washes. In experimental huts, Olyset Duo induced lower levels of oviposition inhibition of surviving wild free-flying blood-fed pyrethroid-resistant *An. gambiae* sl Cove mosquitoes (45% when unwashed and 15% after 20 washes)^14^ compared to what we observed with Royal Guard (83% unwashed and 25% after 20 washes) with the same vector population. The difference in the performance of pyriproxyfen in the two types of pyrethroid-PPF nets could be attributed to one or more of the following effects: to the higher excito-repellent effect of the permethrin insecticide^22,23^ in Olyset Duo which may have prevented mosquitoes from contacting the net long enough to pick up adequate amounts of pyriproxyfen on the net, to the type of pyrethroid-resistant strains tested in laboratory bioassays (VKPR strain with kdr for Olyset Duo versus Cové strain with kdr and metabolic resistance for Royal Guard) or to differences in the surface availability or bleed rate of pyriproxyfen. Chemical analysis results showed that the percentage retention of pyriproxyfen after 20 washes in Royal Guard (57%) was fairly similar to what was reported with Olyset Duo (50%)^14^. Given the improved effects shown by Royal Guard on reproductive suppression and sterility, a greater reduction in clinical malaria across the community might be predicted from Royal Guard than the 12% reduction in clinical incidence shown by Olyset Duo over Olyset Net in the Burkina Faso stepped wedge cluster randomised trial^16^.

Current WHO guidelines require new candidate LLINs which meet efficacy criteria in phase I laboratory and phase II experimental hut studies to demonstrate durability in terms of biological efficacy, net survivorship and fabric integrity ideally for 3 years under operational conditions^17,24^. To assess biological efficacy, nets are destructively sampled from the community over time and tested in WHO cone bioassays and tunnel tests to determine the time point at which bioefficacy is lost using criteria similar to phase I laboratory studies. Results from a durability study of Olyset Duo in Burkina Faso demonstrated that the sterilising effects of Olyset Duo in cone bioassays with pyrethroid-resistant *An. gambiae* sl strains were observed for only 1 month post LLIN distribution; this was however in contrast to entomological data from a parallel cluster randomised trial which found the sterilising effects to last for up to 1 year^25^. While it will be interesting to see how Royal Guard will perform in such durability studies, suitable WHO guidelines and standard operating procedures for monitoring the bioefficacy of pyrethroid-PPF nets in laboratory bioassays are yet to be established. If the improved reproductive suppression over 20 washes shown by Royal Guard is a true proxy for 3 years of field use this would be a major achievement. Given that a host of other factors operate in field conditions other than washing, 3 years bioefficacy should be possible for the pyrethroid component of Royal Guard but may not be achieved by the pyriproxifen component.

To reduce the confounding effects of the pyrethroid component in pyrethroid-PPF nets, mosquito strains tested in bioassays should be pyrethroid-resistant enough to allow sufficient numbers of survivors to assess for the sterilising effects of the pyriproxyfen component. Though we tested a strain which was over 200-fold resistant to pyrethroids (*An. gambiae* sl from Cové, Benin)^18^ in the present study, we however encountered some challenges in the laboratory bioassays which could have confounded the results and therefore should be taken into consideration in bioassay procedures for pyrethroid-PPF nets; (1) the oviposition rates in the control were sometimes very low with the resistant strain (8% in cone bioassays) making the results very difficult to interpret. Dissection of mosquitoes post-exposure to observe and assess deformities in ovary development under microscope^26^ is another option but this is more arduous and requires adequate training. (2) Mosquitoes which were tested unfed failed to take a blood meal post-exposure or were killed in the process. Perhaps uptake of the insecticides during exposure affected the mosquitoes’ ability to take a blood-meal. By contrast, mosquitoes which were blood-fed before exposure provided clear results on reproductive suppression. (3) Mortality rates and blood-feeding inhibition with Royal Guard were both very high in tunnel tests with the pyrethroid-resistant strain before and after 20 washes leaving almost no blood-fed survivors to observe for the reproductive effects of pyriproxyfen. This was rather unexpected considering that the strain was highly resistant to pyrethroids in susceptibility bioassays. This demonstrates a major challenge in identifying suitable strains for bioassays with pyrethroid-PPF nets. Where reliable LLIN durability bio-efficacy data is unattainable in laboratory bioassays, it could be useful to assess field-collected nets in experimental hut trials where possible since the latter provided more interpretable results with pyrethroid-PPF nets.

Based on the findings from our study, Royal Guard was added to the WHO’s list of pre-qualified vector control products in 2019^27^. However, this listing does not provide operational guidance as to whether Royal Guard will be superior to standard pyrethroid-only nets under field use. This will require a WHO policy recommendation establishing pyrethroid-PPF nets as a new LLIN class based on demonstrated improved epidemiological efficacy compared to a standard pyrethroid-only LLIN^28^. Royal Guard is the first pyrethroid-PPF LLIN to be submitted to the WHO's Vector Control Advisory Group (VCAG) on new vector control tools for the assessment of improved epidemiological efficacy^29^. It is currently being evaluated in two large scale community randomised controlled trials; one in East Africa (Tanzania) and another in West Africa (Benin)^29,30^. Studies to assess its durability under operational use over three years have also been integrated within these trials.

## Conclusion

Royal Guard fulfilled existing WHO criteria for efficacy and wash-resistance in laboratory and experimental hut studies and showed superiority over pyrethroid-only LLINs in terms of its ability to sterilise surviving pyrethroid-resistant mosquitoes through the pyriproxyfen component while also inducing mortality and preventing mosquito feeding through the effect of the alpha-cypermthrin component. Royal Guard has the potential to improve malaria vector control and provide better protection against clinical malaria compared to standard pyrethroid-only LLINs in areas where vectors have developed high resistance to pyrethroids. Standardised guidelines and efficacy criteria for evaluating pyrethroid-PPF nets in laboratory assays and durability bioefficacy studies are urgently needed.

## Supplementary information

Supplementary Figure S1

Supplementary Table S1

## Data Availability

The datasets used and/or analysed during the current study are available from the corresponding author on reasonable request.

## Abbreviations

LLIN: Long-lasting insecticidal nets
IRS: Indoor residual spraying
WHO: World Health Organisation
PQ: Prequalification team
VCAG: Vector Control Advisory Group
GPIRM: Global Plan for Insecticide Resistance Management
WHOPES: WHO Pesticide Evaluation Scheme
PPF: Pyriproxyfen
DCT: Disease control technologies USA
CREC: Centre de Recherche Entomologique de Cotonou
LSHTM: London School of Hygiene & Tropical Medicine
Kdr: Knock down resistance
