# Supplementary figures and images for: Efficacy of Royal Guard, a new alpha-cypermethrin and pyriproxyfen treated mosquito net, against pyrethroid-resistant malaria vectors
Source: Sci Rep. 2020 Jul 22;10:12227. doi: 10.1038/s41598-020-69109-5 (PMC7376134; doi:10.1038/s41598-020-69109-5)

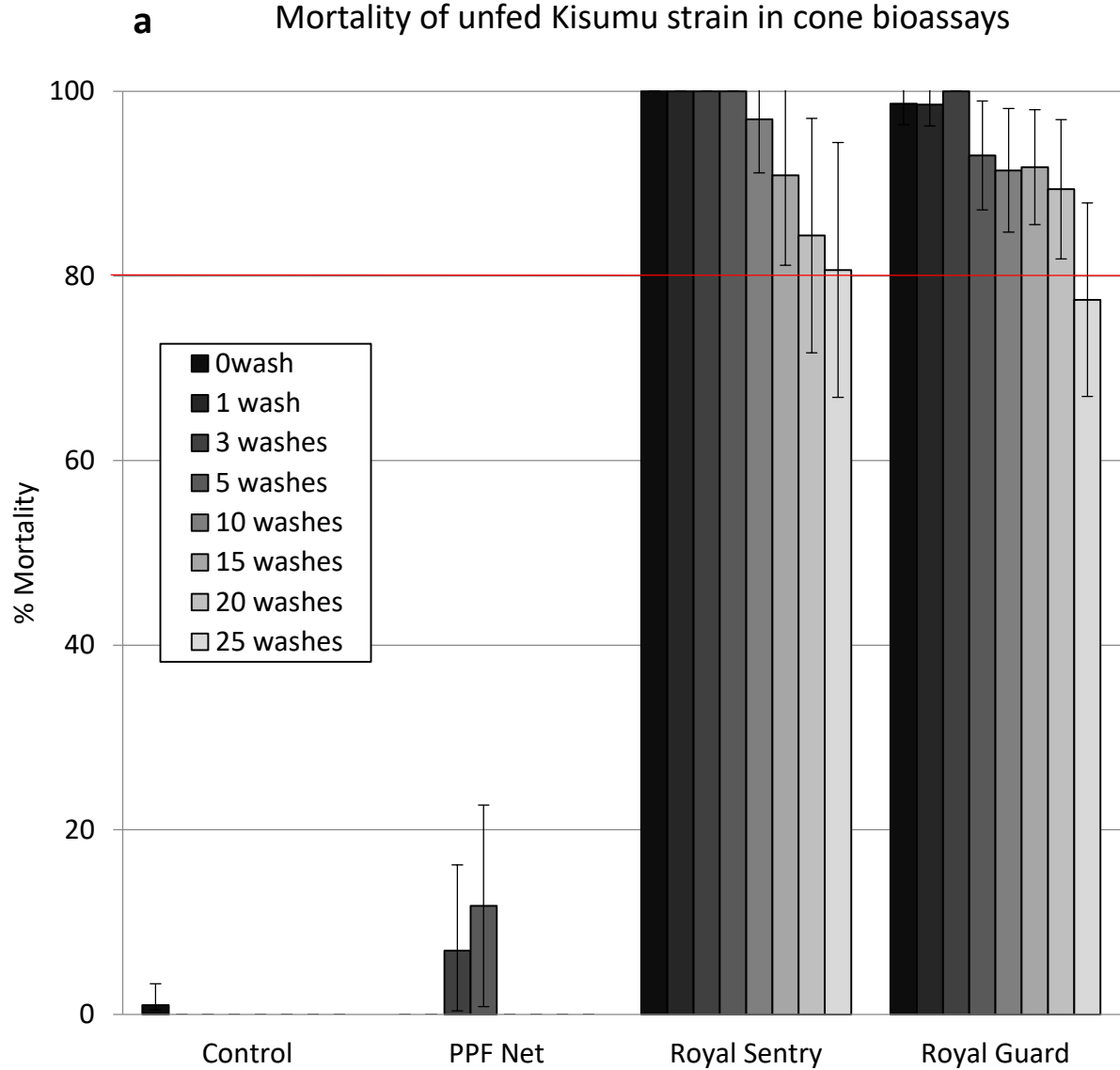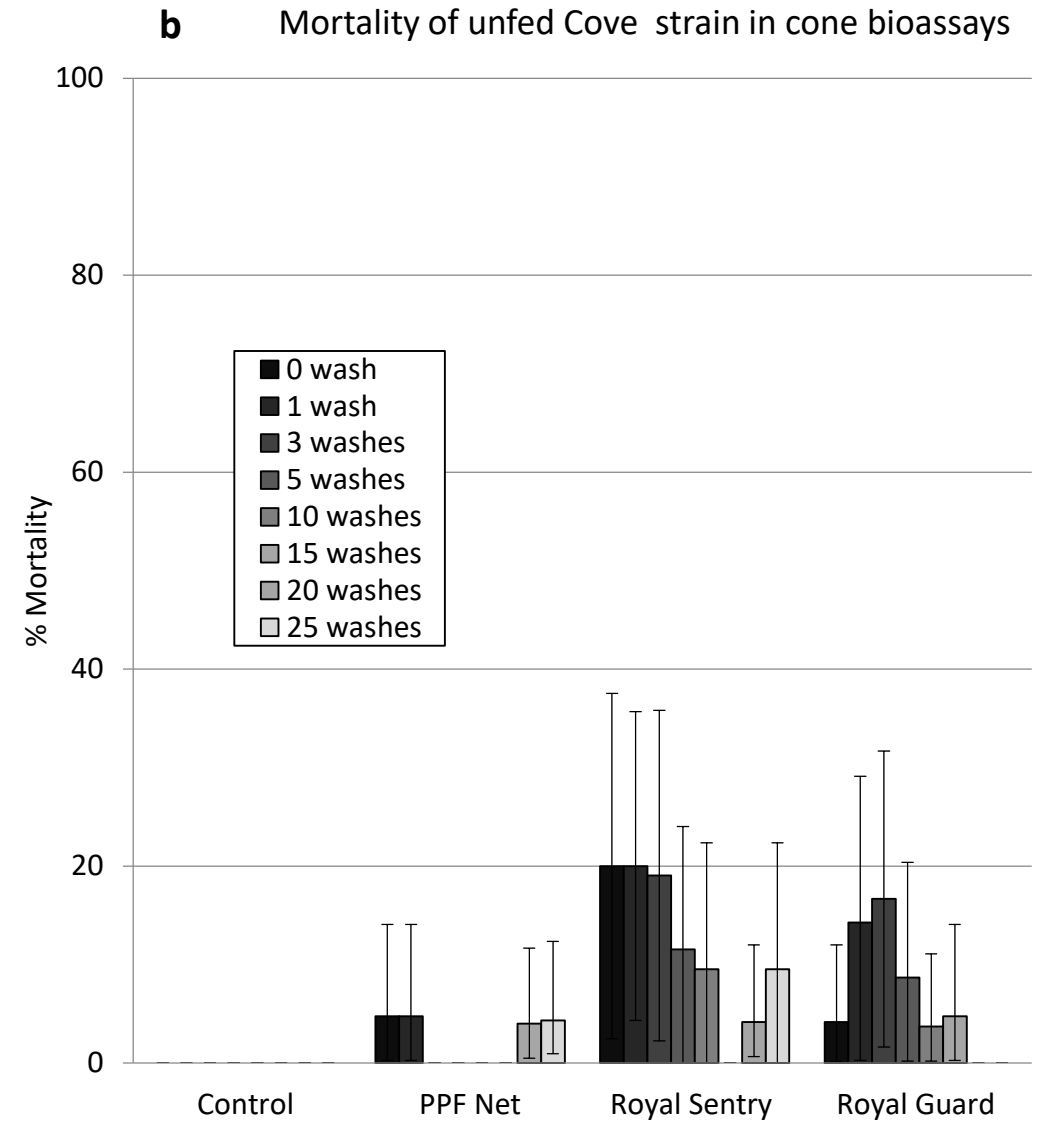

Supplement: Supplementary file 1 — Supplementary Figure S1 [file 41598_2020_69109_MOESM1_ESM.pdf]
