# Supplementary material for: Efficacy of Royal Guard, a new alpha-cypermethrin and pyriproxyfen treated mosquito net, against pyrethroid-resistant malaria vectors
Source: Sci Rep. 2020 Jul 22;10:12227. doi: 10.1038/s41598-020-69109-5 (PMC7376134; doi:10.1038/s41598-020-69109-5)
Supplement: Supplementary file 2 — Supplementary Table S1 [file 41598_2020_69109_MOESM2_ESM.docx]

**Efficacy of Royal Guard, a new alpha-cypermethrin and pyriproxyfen treated mosquito net, against pyrethroid-resistant malaria vectors.**

Corine Ngufor^1,2,3*^

corine.ngufor@lshtm.ac.uk

***corresponding author**

Abel Agbevo^2,3^

Email: aagbevo@crec-lshtm.org

Josias Fagbohoun^2,3^

Email: jfagbohoun@crec-lshtm.org

Augustin Fongnikin^2,3^

afongnikin@crec-lshtm.org

Mark Rowland^1,3^

Email: mark.rowland@lshtm.ac.uk

^1^London School of Hygiene and Tropical Medicine (LSHTM), London, UK

^2^Centre de Recherches Entomologiques de Cotonou (CREC), Benin

^3^Pan African Malaria Vector Research Consortium (PAMVERC), Benin

**Key words:** *experimental huts, Royal Guard, Pyriproxyfen, oviposition, alpha-cypermethrin, LLIN, long-lasting insecticidal net, pyrethroid-PPF, mixture LLIN, pyrethroid resistance, Next-generation LLIN, Anopheles, Cove.*

**Supplementary table information**

Table S1: Detailed results for wild free-flying pyrethroid-resistant *An. gambiae* sl mosquitoes in experimental huts in Cove, Benin

| Net type | Control net | PPF Net | | Duranet | | Royal Guard | |
| --- | --- | --- | --- | --- | --- | --- | --- |
| No of washes | - | 0 | 20 | 0 | 20 | 0 | 20 |
| Total females caught | 1394 | 1247 | 1296 | 731 | 854 | 1339 | 1262 |
| Average catch per night | 26 | 23 | 24 | 14 | 16 | 25 | 23 |
| % Deterrence | - | 11 | 7 | 48 | 39 | 4 | 10 |
| Total Exiting | 603 | 494 | 386 | 391 | 400 | 775 | 589 |
| % Exiting | 43 | 40 | 30 | 53 | 47 | 58 | 47 |
| 95% Conf Interval | (41-46) | (37-42) | (27-32) | (50-57) | (43-50) | (55-61) | (44-49) |
| % Inside Net | 33 | 39 | 51 | 24 | 34 | 18 | 34 |
| Total blood-fed | 690 | 693 | 909 | 238 | 392 | 312 | 570 |
| Blood-feeding (%) | 50 | 55.6 | 70.1 | 33 | 46 | 23.3 | 45 |
| 95% Conf Interval | (47-52) | (53-58) | (68-73) | (29-36) | (43-49) | (21-26) | (43-46) |
| Blood-feeding inhibition (%) | - | 0 | 0 | 34 | 7 | 53 | 10 |
| 95% Conf Interval | - | - | - | (31-38) | (6-9) | (50-56) | (9-11) |
| No. dead after 24h | 34 | 95 | 79 | 142 | 96 | 301 | 166 |
| % dead after 24h | 2 | 8 | 6 | 19 | 11 | 22 | 13 |
| % Corrected Mortality | - | 5.3 | 3.8 | 17.4 | 9 | 21 | 11 |
| 95% Conf Interval | - | (4-7) | (3-5) | (15-20) | (7-11) | (18-23) | (9-13) |
| Personal Protection (%) | - | 0 | 0 | 66 | 43 | 54 | 17 |
| Killing effect (%) | - | 4 | 3 | 8 | 4 | 19 | 9 |
